# Evaluation of preclinical efficacy of everolimus and pasireotide in thyroid cancer cell lines and xenograft models

**DOI:** 10.1371/journal.pone.0206309

**Published:** 2019-02-26

**Authors:** Taofeek K. Owonikoko, Guojing Zhang, Shenila B. Lallani, Zhengjia Chen, Deborah E. Martinson, Fadlo R. Khuri, Sagar Lonial, Adam Marcus, Shi-Yong Sun

**Affiliations:** 1 Department of Hematology & Medical Oncology, Emory University School of Medicine, Atlanta, Georgia, United States of America; 2 Winship Cancer Institute of Emory University, Atlanta, Georgia, United States of America; 3 Department of Biostatistics and Bioinformatics, Rollins School of Public Health, Emory University, Atlanta, Georgia, United States of America; Bauer Research Foundation, UNITED STATES

## Abstract

**Background:**

Signaling through mTOR and somatostatin pathway is implicated in thyroid cancer development.

**Method:**

We evaluated everolimus, an mTOR inhibitor and pasireotide, a multi receptor somatostatin analogue as potential therapy of thyroid cancer focusing on the *in vitro* and *in vivo* efficacy, as well as possible mechanism to explain any observed interaction.

**Results:**

Both everolimus and pasireotide inhibit the growth of thyroid cancer cell lines *in vitro* with varied efficacy that correlates with tumor origin and somatostatin receptor (SSTR) expression profile of the cell lines. *In vitro* activity of everolimus show positive correlation with the expression of SSTR types 1, 4 and 5 (CC: 0.9; 0.85, 0.87) while pasireotide activity show negative correlation with SSTR2 (CC: -0.87). Although there is greater modulation of pS6 when pasireotide is combined with everolimus, there is no significant abrogation of the expected feedback upregulation of AKT induced by everolimus. Also, the combination is not significantly better than each agent alone in short and long term *in vitro* assays. Continuous administration of everolimus at a low dose as opposed to high intermittent dosing schedule has greater antitumor efficacy against thyroid cancer xenografts *in vivo*. Pasireotide LAR has modest in vivo efficacy and the combination of everolimus and pasireotide LAR achieve greater tumor growth inhibition than each agent alone in TPC-1 xenograft model of thyroid cancer (p = 0.048).

**Conclusion:**

Our findings provide support for the clinical evaluation of everolimus and pasireotide in thyroid cancer and other neuroendocrine tumors.

## Introduction

There is an increasing incidence of thyroid cancers in the US and worldwide with approximately 200% increase in the last 3 decades and more than 62000 new diagnoses estimated in the US in 2015 [1, 2]. While the majority of patients are potentially curable with surgery, up to a 3^rd^ of the patients suffer local or distant disease recurrence on long term follow-up. The effective treatment of relapsed patients especially those with iodine refractory disease remains a major management challenge.

Somatostatin receptor (SSTR) mediated signaling has been shown to result in anti-proliferative effect and cytotoxicity against benign tumors such as pheochromcytopams as well as cancer cell lines [3–5]. This effect is mediated in part through the inhibition of PI3K/AKT signaling pathway, which is upstream of mTOR intracellular signaling cascade. Similar to other endocrine tumors, various SSTR subtypes are frequently expressed in normal and malignant thyroid epithelial cells where it has inhibitory effect on cell growth and function [6–12]. Aberrant TSH stimulated signaling in thyroid cancers is key to the increased proliferation and survival [13]. This proliferative effect is also mediated principally through the mTOR signaling pathway [13]. Due to the remarkable plasticity of cancer cells and the co-occurrence of several independent but interconnected deregulated signaling aberrations across various pathways, concurrent inhibition of several signaling pathways appears essential for optimal clinical efficacy of targeted agents. This recognition has fueled the emerging paradigm in targeted therapy of solid tumors of maximal pathway inhibition through the use of multiple targeted inhibitors with complementary activity to disrupt signaling cascades at multiple critical nodes along the signaling pathway [14–16]. Such an approach is expected to be more effective in abrogating the signaling aberration while at the same time prevent the development of alternative bypass signaling as a resistance mechanism by the cancer cell to overcome therapeutic efficacy. Because of the signaling convergence of both somatostatin receptor and mTOR pathway and the implication of both pathways in thyroid cancer, a therapeutic strategy of combined somatostatin analogue therapy and mTOR inhibition is likely to achieve improved efficacy. The potential of such a therapeutic approach has been demonstrated in some neuroendocrine tumors as well as in medullary variant of thyroid cancer but not yet in thyroid cancer of follicular epithelium origin [17, 18]. Because of the differential pattern of SSTR receptor between different endocrine tumors, careful evaluation of the potential success of such a strategy in the preclinical models of thyroid cancer will be useful to guide future clinical development for this disease where new options of therapy are needed.

This preclinical study was designed to evaluate the single agent and combined *in vitro* and *in vivo* anticancer activities of everolimus, an established mTOR inhibitor and pasireotide, a somatostatin analogue, with a broad affinity spectrum similar to somatostatin, the naturally occurring ligand [19, 20].

## Materials and methods

### Reagents

Everolimus, pasireotide (SOM230), pasireotide LAR and matching placebo LAR were provided by Novartis Oncology under a material transfer agreement with Emory University. Everolimus was dissolved in DMSO, aliquoted and stored at -20°F until ready for use for *in vitro* experiments and prepared fresh in PBS for xenograft experiments. Treatment grade samples of pasireotide and pasireotide LAR along with matching placebo were employed as provided for *in vitro* and *in vivo* experiments. The following antibodies were employed at the indicated dilutions for Western Blot assays: actin (Sigma-Aldrich, St. Louis, MO Cat# A2066) at 1:3000 dilution; S6 (Cell Signaling, Danvers MA Cat#2217) at 1:1000 dilution, phospho-S6^Ser235/236^ (Cell Signaling, Danvers MA Cat#2211) at 1:1000 dilution, Akt (Cell Signaling, Danvers MA Cat#9272) at 1:1000 dilution, phospho-Akt^Ser473 (736E11)^ (Cell Signaling, Danvers MA Cat#3787) at 1:2000 dilution, eIF4E (Cell Signaling, Danvers MA Cat#9742) at 1:1000 dilution, phospho-eIF4E^Ser209^ (Cell Signaling, Danvers MA Cat#9741) at 1:1000 dilution, 4E-BP1 (Cell Signaling, Danvers MA Cat#9452) at 1:1000 dilution, phospho-4E-BP1^Thr37/46^ (Cell Signaling, Danvers MA Cat#2855) at 1:1000 dilution, mTOR (Cell Signaling, Danvers MA Cat#2972) at 1:1000 dilution, phospho-mTOR^Ser2448 (49F9)^ (Cell Signaling, Danvers MA Cat#2976) at 1:1000, cleaved caspase 3 (Cell Signaling, Danvers MA Cat”96615) 1:500; caspase 3 (IMGENEX; USA; Cat #IMG-144A) at 1:500 and somatostatin receptors 1, 2, 3, 4 and 5(Abcam, Cambridge, MA USA) at 1:200; 1:500; 1:5000, 1:1000 and 1:1000 respectively.

### Cell lines and cell culture

Thyroid cancer cell lines validated to be of thyroid origin[21] were generously provided by Rebecca Schweppes, PhD University of Colorado, Denver, CO (TPC-1, BCPAP, CAL-62) and Prof. Nils-Erik Heldin, Uppsala University, Stockholm Sweden (C643, U-HTh7, U-HTh74-cl.7). We employed the MycoAlertTM Mycoplasma Detection Kit (Lonza Inc. Allendale, NJ 07401 USA; Cat# LT07-118) to test the cell lines for mycoplasma contamination prior to the described experiments. BCPAP and TPC1 represent differentiated thyroid cancer harboring BRAF V600E and RET/PTC1 mutations respectively. Cal-62, C643, U-HTh7 and U-HTh74-cl.7 were derived from patients with undifferentiated thyroid cancer and are all wild type for BRAF whereas C643 harbor HRAS (G13R) mutation.[21] Cells were grown as monolayer culture in RPMI 1640 medium (TPC-1, BCPAP, CAL-62, C643) or MEM medium (U-HTh7, U-HTh74-cl.7) supplemented with up to 5–10% fetal bovine serum at 37 °C under humidified condition of 5% CO_2_ and 95% air.

### Short-term growth inhibition assay

Short term cytotoxicity assay was employed to establish the single agent cytotoxicity of everolimus and pasireotide against thyroid cancer cell lines. Cells were cultured in 96-well cell culture plates and treated with the indicated agents singly and in combination in the exponential growth phase by continuous drug exposure starting 24 hours after seeding. Surviving viable cell number was detected using the sulforhodamine B (SRB) assay, according to the manufacturer’s recommendation (Sigma-Aldrich, St. Louis, MO). Briefly, drug-containing medium was discarded followed by fixation of the adherent cells with trichloroacetic acid (10% w/v) for 60 minutes at 4°C. The fixation step was followed by washing in deionized water up to 5 times after which the plates were left to dry in ambient air. Subsequently, each well was filled with 50uL SRB reagent solution (0.4% w/v in 1% acetic acid) and incubated for 10 minutes at room temperature. After removing unbound SRB reagent by washing with 1% acetic acid, plates were air-dried and bound stain was solubilized wit 100uL of 10mM unbuffered Tris base (pH 10.5). Optical density of the well was read using a microplate reader at 492 nM. Each tested concentration was analyzed from six replicate wells. The percentage of growth inhibition Short term SRB assay: Freshly thawed cell aliquots were seeded in 96-well plates at approximately 1–2 x 10^3^ cells per well.

### Long-term colony formation assay

This assay was performed as we previously described [22]. Cells were seeded in 12-well culture plates at a density of approximately 100 cells per well. Following overnight growth and cell attachment, drug-free culture medium was replaced with DMSO-containing medium only (control) or medium containing everolimus or pasireotide. Each condition was tested in triplicate wells. The medium was replaced every 3–5 days until plates were ready for colony count (typically 10 to 14 days after seeding). Cells were fixed by exposure to 70% methanol for 5 minutes, repeated once. This was followed by staining with 0.1% crystal violet for 5 min and excess stain was washed off under running tap water after which the plates were air-dried. The number of distinctly stained colonies (containing at least 50 cells per colony) was counted using a colony counter (Fisher Scientific) and the results averaged for each treatment group.

### In vivo tumor growth inhibition

In vivo growth inhibition has been shown to be representative of expected activity of anticancer agents in human. Therefore, we evaluated the ability of everolimus and pasireotide LAR alone and in combination to inhibit tumor growth using standard subcutaneous xenograft models generated using representative cell lines. Animal experiments were conducted in compliance with humane treatment of research animals under an experimental protocol approved by the Institutional Animal Care and Use Committee (IACUC) of Emory University (IACUC approval number: DAR-2002527-060417BN). Tumor xenografts for the initial experiments were raised in 3–4 week old female Harlan Nude rats (Hsd:RH-Foxn1(rnu/rnu; Harlan, IN) out of concern for cross specificity of somatostatin analogue. Subsequent experiments were conducted in 6-week old female SCID mice (Harlan Industries, Indianapolis, IN). Animals were housed under pathogen-free conditions in microisolator cages and fed with laboratory chow and water *ad libitum*. Representative cell lines (TPC-1, BCPAP) with different *in vitro* growth characteristics were employed for the *in vivo* animal experiments. BCPAP (6 x 10^7^) and BCPAP (2 x 10^7^) suspended in serum-free medium were injected subcutaneously into the flank region of nude mice. Tumor growth was monitored and measured by caliper 2–3 times per week. When the tumors achieved a volume of approximately 100 mm^3^ using the formula: [(length x width^2^)/2], groups of tumor-bearing mice (approximately 6 mice per group) were matched for body weight and tumor volume and randomly assigned to treatments: vehicle, everolimus (1mg/kg orally daily, 2.5mg/kg intermittently thrice weekly and 5mg/kg intermittently thrice weekly), pasireotide LAR vehicle, pasireotide LAR (10mg/kg and 20mg/kg subcutaneously once on day 1). Animal treatment started approximately 30 days following cell inoculation. At the end of the experiments, subcutaneous tumors were harvested and weighed following animal sacrifice by cervical dislocation.

### Western blot analysis

Whole cell protein lysates and Western blot analysis were performed according to standard procedure in our lab as we have described previously [23].

### Detection and quantification of somatostatin receptor expression by immunofluorescence microscopy

We employed immunofluorescence microscopy to detect the level of expression of the five different somatostatin receptors (SSTR) on the cell lines employed for this preclinical work. Cells grown on glass cover slips for 24 hours were fixed and processed for immunofluorescence microscopy as previously described. Cells were stained using primary antibodies against the five different receptor subtypes, SSTR1, SSTR2, SSTR3, SSTR4, SSTR5 and secondary Alexa 488-conjugated goat anti-mouse IgG. Coverslips were mounted onto slides and imaged using Zeiss LSM510 META confocal microscope with a 40X Plan-NEOFLUAR oil objective (NA 1.3).

## Results

### Everolimus and pasireotide variably inhibit thyroid cancer cell lines growth in vitro

**Fig 1:**
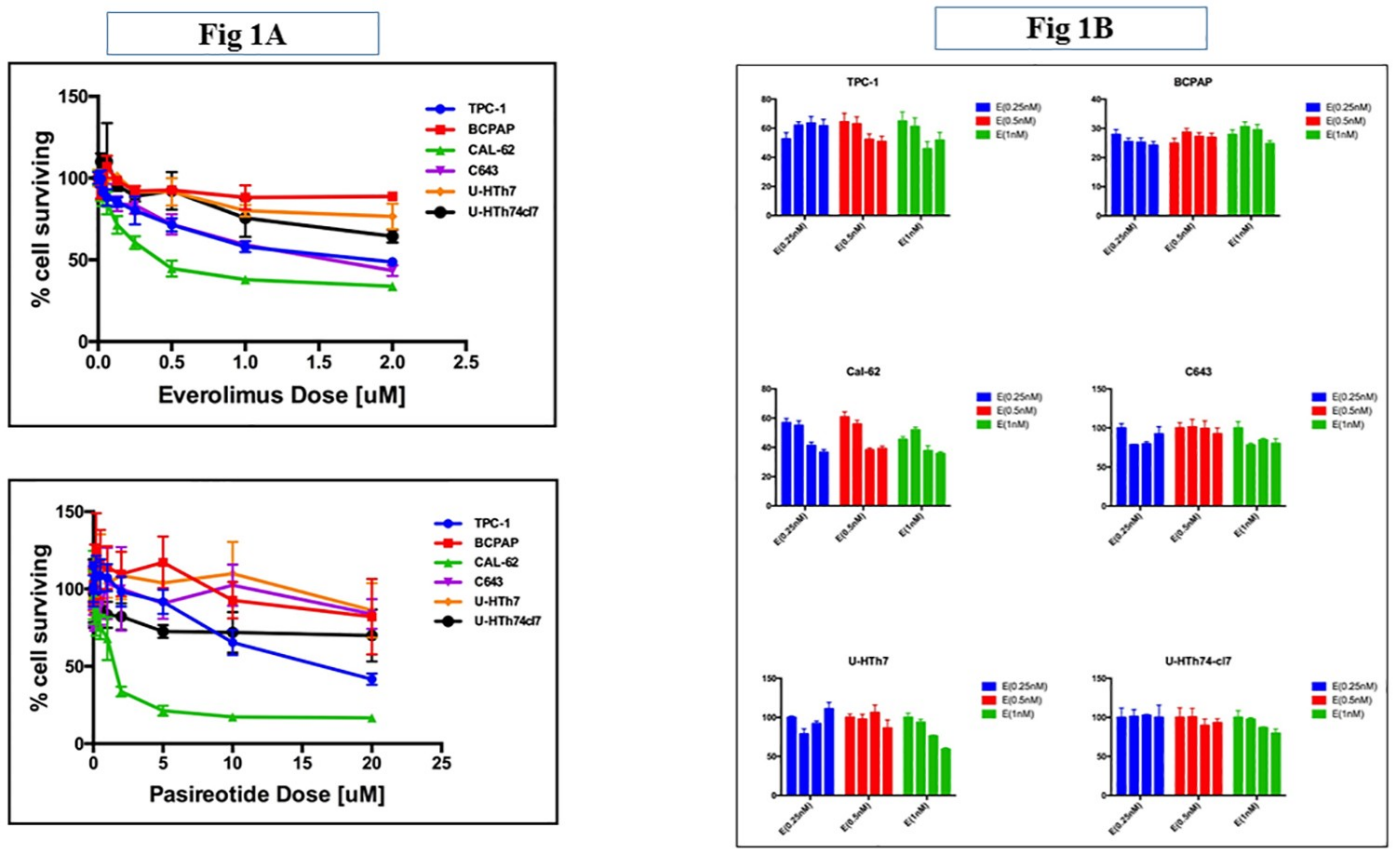
Everolimus and pasireotide inhibit growth of thyroid cancer cell lines in short term (A) and long term in vitro cytotoxicity assays (B). A, Human thyroid cancer cell lines derived from well-differentiated (TPC-1 and BCPAP), poorly-differentiated (Cal-62) and anaplastic (C643, U-HTh-7 and U-HTh-74.cl) thyroid cancer were seeded in 96-well plates and allowed to grow overnight. Exponentially growing cells were treated the next day with serially increasing concentrations of pasireotide (0.01 to 20μM) and everolimus (0.01 to 2μM). After 72 hours of continuous drug exposure, cell numbers were estimated using the SRB assay. IC_50_ concentration was estimated from the growth inhibition curves for pasireotide (top) and everolimus (bottom) using GraphPad prism software. B, Cells plated in 12-well plates (50 cells per well) were treated in triplicates with vehicle (C), everolimus (E, 0.25, 0.5 and 1nM), pasireotide (fixed dose of 0.5μM) and the combination (E+P). Drug containing medium was refreshed every 3–5 days for up to 14 days. The number of distinct colonies formed at the end of the experiment was counted by visual observation following Crystal Violet staining and presented as the mean of 3 independent experiments (representative colony culture plates presented as supplemental data); bar graphs represent control, everolimus, pasireotide and the combination (respectively) for varying concentrations of everolimus (0.25, 0.5 and 1nM).

To determine the growth inhibitory effect of everolimus, we treated six thyroid cell lines representative of well differentiated, poorly differentiated and anaplastic histologies using concentrations ranging between 2nM and 1 μM. Cells were treated by continuous exposure for 3 days and surviving cell fraction was quantified by SRB assay. Everolimus showed a concentration dependent reduction in surviving cell fraction with IC_50_ ranging between 0.62nM and 32.38nM (Fig 1A and Table 1). To determine the growth inhibitory effect of pasireotide, thyroid cancer cell lines were treated with serial dilution of pasireotide at concentrations ranging between 1nM and 20 μM. Cells were treated by continuous exposure for 3 days and surviving cell fraction was quantified by SRB assay. Pasireotide showed overall minimal toxicity in short-term cytotoxicity assay at clinically relevant concentrations (Fig 1A).

**Table 1:** Intensity of surface expression of somatostatin receptor subtypes expressed in arbitrary Fluorescence Intensity Counts in thyroid cancer cell lines measured by immunofluorescence microscopy and IC50 concentrations for pasireotide and everolimus in thyroid cancer cell lines.

Cell Line	SSTR1	SSTR2	SSTR3	SSTR4	SSTR5	Everolimus IC_50_ [nM]	Pasireotide IC_50_ [μM]
TPC-1	140	967	116	155	486	7.57	7.83
BCPAP	231	556	189	156	177	2.19	8.65
Cal-62	460	1240	247	395	310	0.99	28.61
C643	301	805	327	405	658	0.62	40.69
U-HTh-7	270	396	267	290	376	4.436	13.24
U-HTh-74.cl	930	900	230	679	874	32.38	16.61
Mean Expression	389	811	229	347	480		
Correlation coefficients with Everolimus IC_50_	0.90	0.22	0.06	0.85	0.87		
Correlation coefficients with Pasireotide IC_50_	-0.20	-0.87	-0.04	-0.31	-0.39		

In order to exclude assay dependent effect and to evaluate for longer-term cytotoxicity, we employed colony formation assay to assess the effect of pasireotide and everolimus against representative thyroid cancer cell lines. Seeded cell lines were exposed to clinically relevant concentrations of everolimus (0.25nM, 0.5nM and 1nM) along with a fixed concentration of pasireotide (0.5μM) and the combination. There was minimal activity of everolimus at the concentrations tested while pasireotide showed modest activity especially against the undifferentiated thyroid cancer cell lines (Fig 1B). The colony formation assay did not show any significant increase in cytotoxicity with the combination over each agent alone (Fig 1B **and** S1 Fig).

### Cytotoxicity of everolimus and pasireotide correlates with somatostatin receptor expression

**Fig 2:**
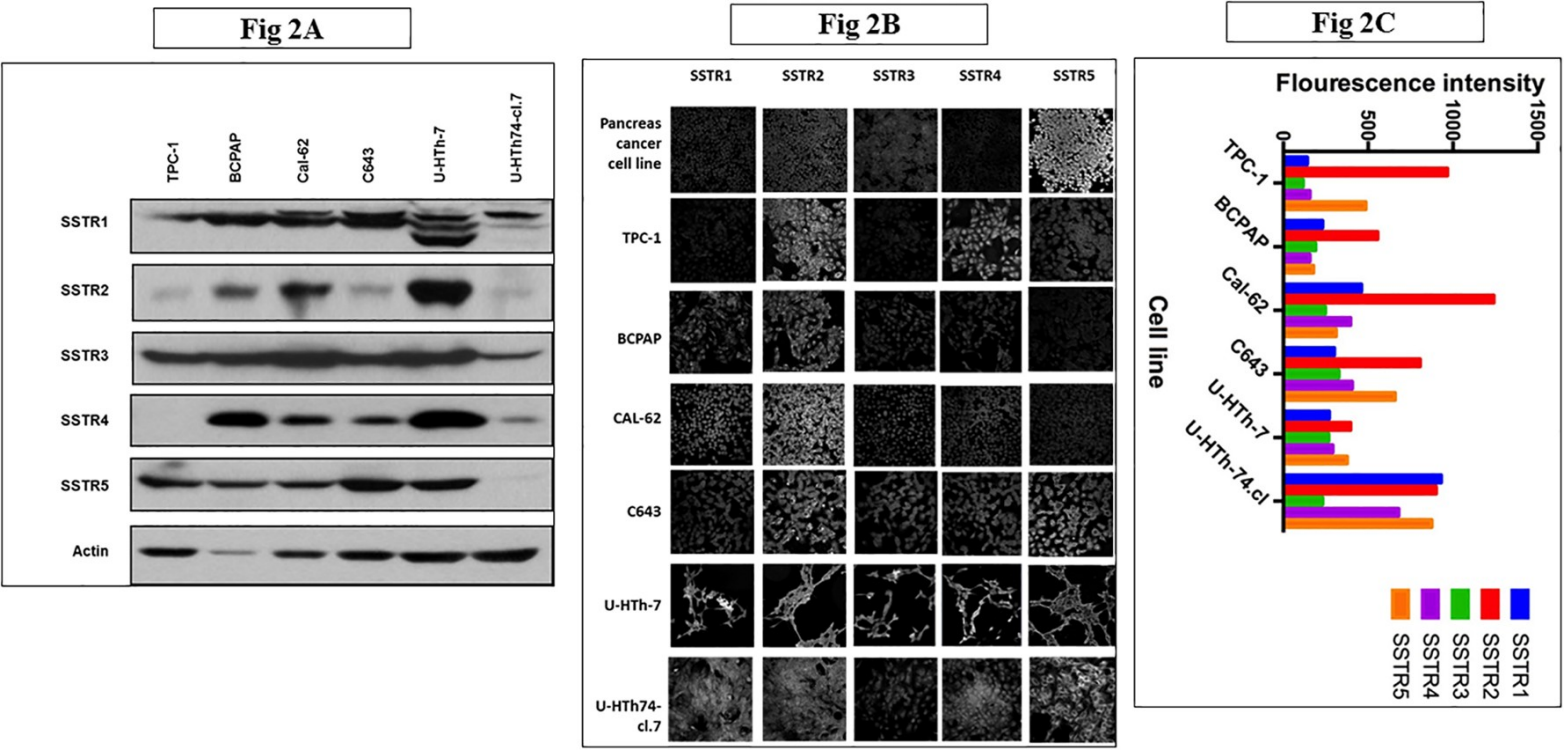
Total cellular expression (A) and membrane expression (B and C) of somatostatin receptors in thyroid cancer cell lines. A, Whole-cell protein lysates prepared from the six thyroid cancer cell lines were employed for Western blot analysis and probed with specific primary antibodies to the five different subtypes of SSTR. The immunoblots show differential expression of SSTR1, 2, 3, 4 and 5. B and C, Intact membrane expression of SSTR1, 2, 3, 4 and 5 was detected (B) and quantified (C) by automated intensity counting on immunofluorescence microscopy using Zeiss LSM510 META confocal microscope with a 40X Plan-NEOFLUAR oil objective (NA 1.3).

Pasireotide has affinity against SSTR1, 2, 3 and 5 subtypes, a feature that potentially makes it more effective than earlier generations of somatostatin analogues whose affinity is restricted to only a subset of the SSTR. Furthermore, downstream signaling following binding of somatostatin to SSTR cascades in part through the PI3K/Akt/mTOR pathway. In order to evaluate whether activity of pasireotide and everolimus is dependent on SSTR expression, we determined the degree of expression of the five SSTR subtypes in the six thyroid cancer cell lines. Total receptor expression was determined by Western blot assay as previously described. We noted variable receptor expression with a generally higher intensity of SSTR staining in the undifferentiated and anaplastic cancer cell lines compared to the well-differentiated cell lines (Fig 2A **and Figures A, B, C, D & E in** S1 File).

We also assessed surface receptor expression using immunofluorescence to detect and quantify native surface expression of all five receptor subtypes where they can be bound by pasireotide. Consistent with the Western Blot result, there was variable expression of SSTR in this panel of thyroid cancer cell lines (Fig 2B). The intensity of expression was stronger in the undifferentiated/anaplastic cell lines as compared to the well-differentiated cell lines (Fig 2C). We observed a positive correlation between the everolimus activity and the degree of expression of SSTR 1, 4 and 5 as measured by immunofluorescence (Table 1).

Conversely, pasireotide activity (measured as IC_50_ concentration) showed a modest but negative correlation with SSTR expression. Everolimus was strongly correlated with SSTR1, 4 and 5 expression while pasireotide correlation was strongest with SSTR1 and 2.

### Pasireotide prevents everolimus-induced pAKT reactivation in sensitive cell lines

**Fig 3:**
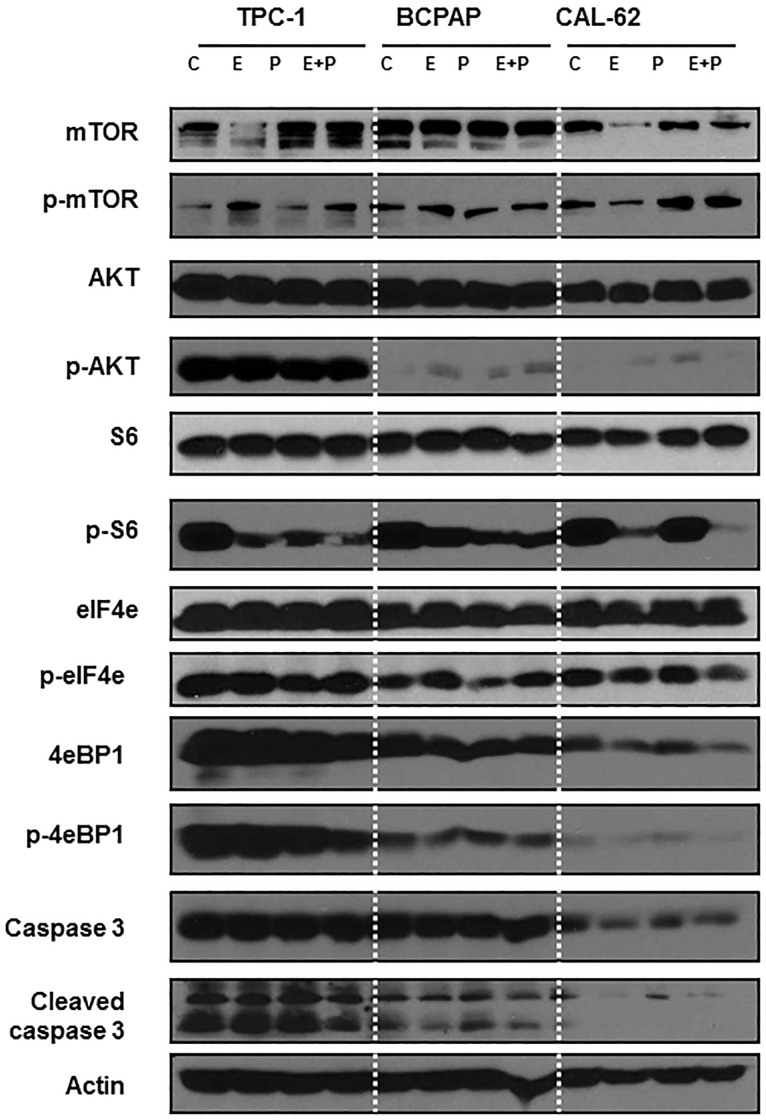
PI3k/mTOR pathway modulation by everolimus, pasireotide singly and in combination. Cell lines were treated with vehicle (C), everolimus (1nM) pasireotide (0.5μM), and their combination for 24 h. Whole-cell protein lysates prepared from harvested cell lines were employed for Western blotting to detect various component proteins that are critical mediators of the PI3K/AKT/mTOR pathway signaling cascade.

A feedback reactivation of the mTOR signaling cascade through the unopposed action mTORC2 complex leading to Akt activation has been well described as an escape mechanism from mTORC1 predominant inhibitory action of everolimus. Thusly, the combination of pasireotide and everolimus is expected to delay or overcome this resistance mechanism through the potential inhibition of PI3K/Akt activity by pasireotide. We therefore sought to explore whether such cooperative interaction is demonstrable in cell lines treated with pasireotide and everolimus. We employed representative cell lines with poor sensitivity (TPC-1), intermediate sensitivity (BCPAP) and high sensitivity (Cal-62) to everolimus to interrogate the degree of modulation of the key signal relay nodes in the PI3/AKT/mTOR signaling pathway. As expected, everolimus caused a significant reduction in pS6 expression, which was further accentuated with the combination of everolimus and pasireotide in all the cell lines (Fig 3 **and Figures R and S in** S1 File).

Furthermore, the least sensitive cell line showed a very high basal expression of pAKT, p4eBP1 and peIF4e whereas the more sensitive cell lines showed low basal levels. Everolimus induced a modest increase in pAKT in the sensitive cell lines but caused no further increase in pAKT in the least sensitive cell lines. When combined with everolimus, pasireotide further enhanced the reduction in pS6 and p4eBP1 expression but had no significant impact on eIF4e levels. Furthermore, there was only a modest effect of pasireotide in preventing pAKT induction by everolimus (Fig 3 **and Figures F, G, H, I, L, M, N, O, P, Q in** S1 File).

### Everolimus and pasireotide inhibit thyroid cancer xenograft in vivo

**Fig 4:**
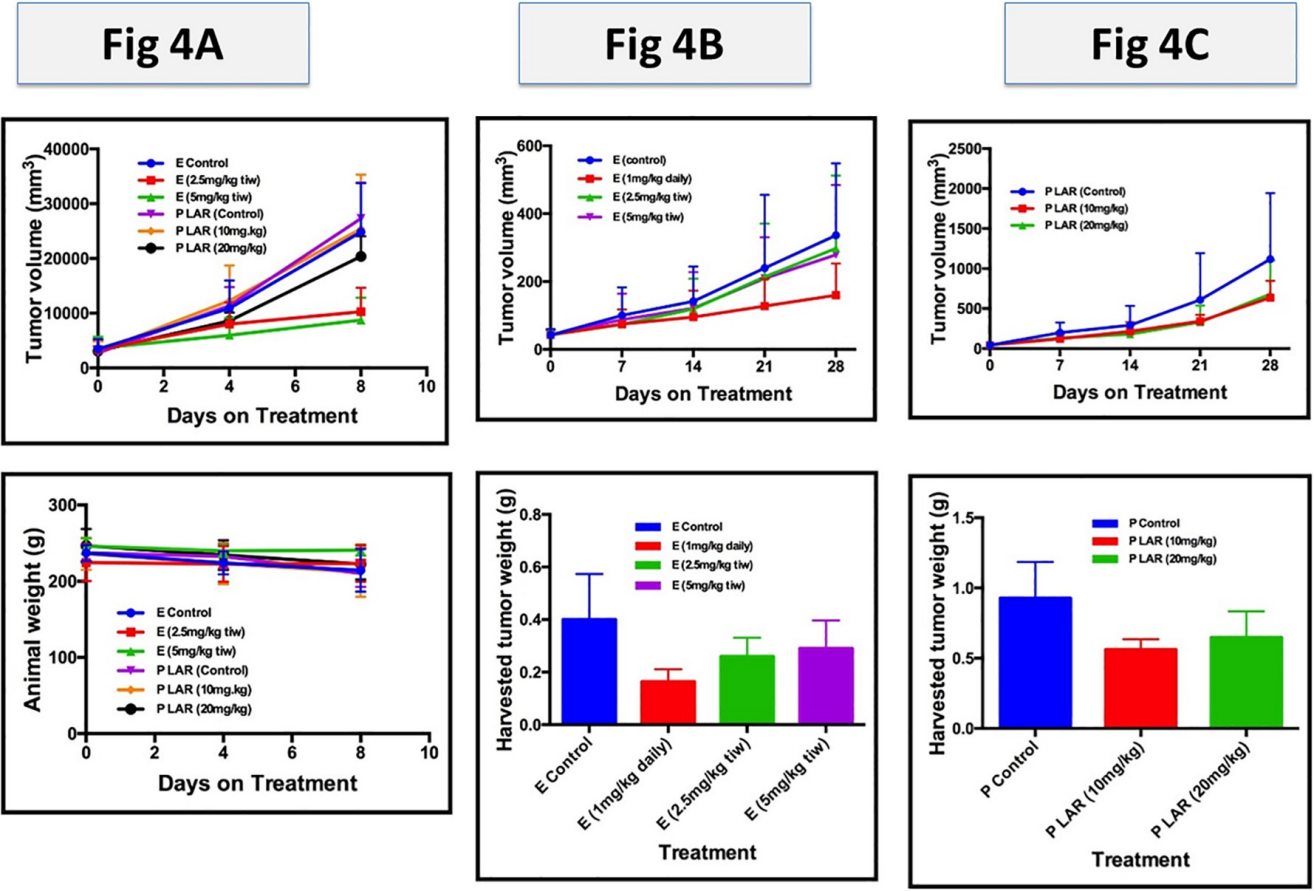
Everolimus efficacy against thyroid cancer xenografts is dose (A) and schedule dependent (B) while pasireotide showed modest efficacy that was not dose dependent (C). A: Dose dependent tumor growth inhibition by everolimus and pasireotide in a TPC-1 tumor xenograft. TPC-1 xenografts raised in nude rats were treated in matched groups of 4–6 tumor-bearing rats with vehicle (E control), everolimus (E, 2.5mg/kg and 5mg/kg, by oral gavage, (o.g) thrice weekly), pasireotide LAR vehicle (P LAR control) and pasireotide (P LAR, 10mg/kg and 20mg/kg subcut once). Tumor sizes and rat body weights were measured twice weekly. Animals were sacrificed on day 9 when the tumor burden reached the threshold established by the IACUC guideline. B: Intermittent versus continuous dosing of everolimus in BCPAP xenograft; and C: Growth inhibition by pasireotide in a BCPAP tumor xenograft. In a separate experiment, BCPAP xenografts raised in nude mice were treated with vehicle (C), everolimus by continuous (1mg/kg, o.g daily) or intermittent (2.5mg/kg and 5mg/kg o.g thrice weekly) dosing, pasireotide vehicle (P control) and pasireotide (10mg/kg and 20mg/kg subcut once). Tumor and animal sizes were measured twice per week.

In order to establish whether the *in vitro* growth inhibitory activity of everolimus and pasireotide against thyroid cancer cell lines will be replicated *in vivo* we tested the efficacy of both compounds against subcutaneous TPC-1 thyroid cancer xenografts in immunodeficient animals. There was a dose dependent tumor growth inhibition observed with everolimus where the higher intermittent dose (5mg/kg) appears more effective than the lower intermittent dose (2.5mg/kg) in the TPC-1 xenograft model (p = 0.07 for C vs. E5; p = 0.1 for C vs. E2.5; Fig 4A).

Compared to control, there is a modest but non-significant growth inhibition by both the low (10mg/kg; p = 0.44) and high (20mg/kg; p = 0.20) doses of pasireotide against TPC-1 (Fig 4A). In a PK/PD animal simulation of pharmacodynamic data obtained from patients, continuous daily dosing of everolimus achieved greater target modulation than the intermittent high dose administration and is the preferred dosing schedule employed for cancer patients in the clinic [24]. In order to extend our findings in TPC-1 and to better simulate the clinical use of everolimus, we subsequently compared the efficacy of continuous dosing with low and high intermittent dosing schedules of everolimus in BCPAP thyroid cancer xenografts. Everolimus dose of 1mg/kg daily continuously achieved greater tumor growth inhibition (p = 0.06) in comparison to everolimus dose of 2.5mg/kg (p = 0.39) or 5mg/kg (p = 0.76) dosed intermittently thrice per week (Fig 4B). We employed this daily dosing schedule for subsequent evaluation of everolimus. Similar to TPC-1 xenograft, there was a modest tumor growth inhibition observed with pasireotide (Fig 4C). This did not reach statistical significance both for the low 10mg/kg dose (p = 0.12) and the high 20mg/kg dose (p = 0.16).

**Fig 5:**
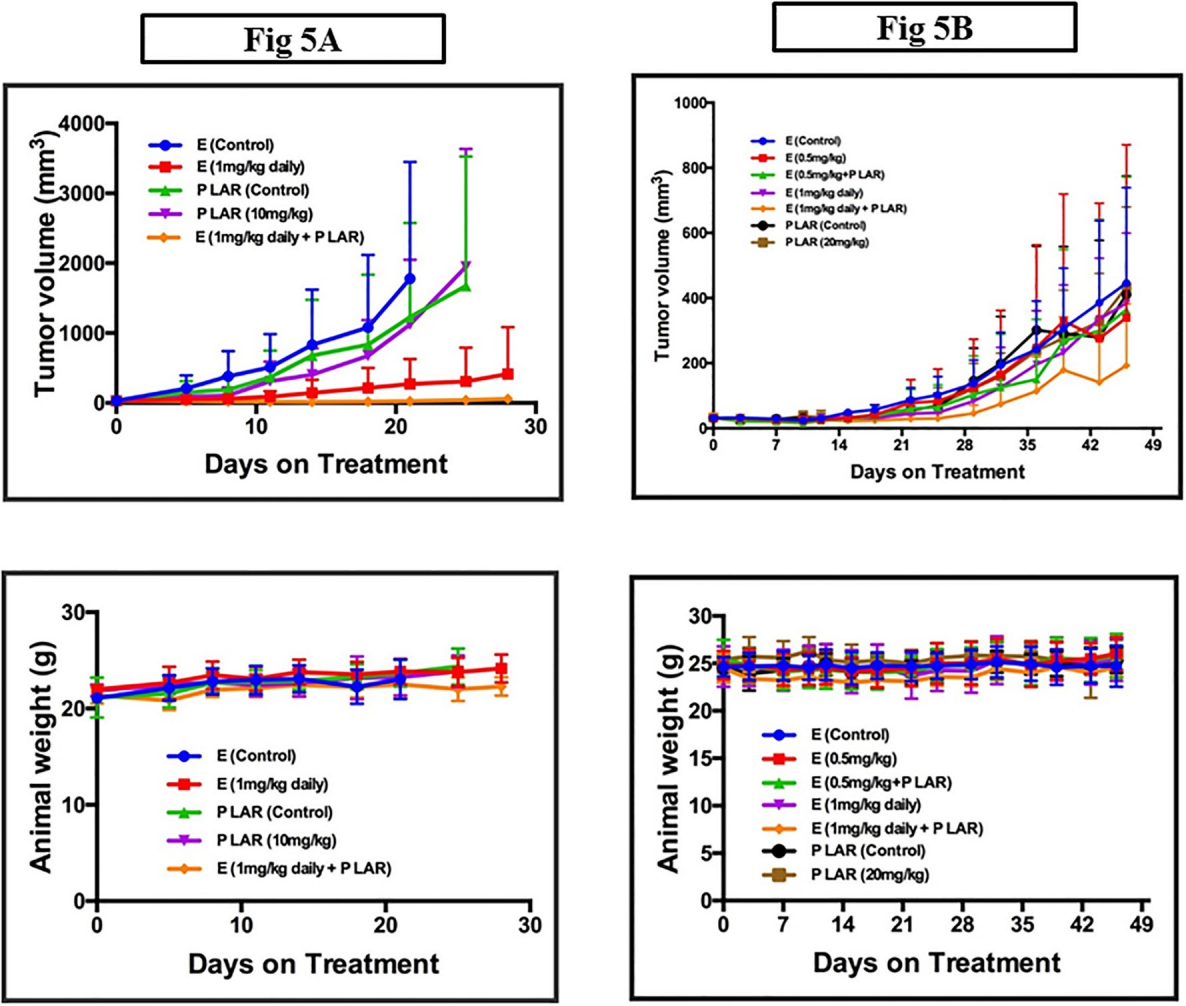
The combination of everolimus and pasireotide showed additive effect in TPC-1 xenografts (A) but not in BCPAP xenograft (B). A: Everolimus and pasireotide in TPC-1 xenograft. Tumor bearing mice were treated in groups of 6 mice with vehicle (E Control), everolimus (E) 1mg/kg o.g. daily continuously, pasireotide LAR vehicle subcut once (P LAR control) and pasireotide LAR 20mg/kg once subcut (P LAR) or the combination of pasireotide and everolimus. Tumor sizes and animal weights were monitored twice weekly as described above. B: Everolimus and pasireotide in BCPAP xenograft. Tumor bearing mice were treated in groups of 6 mice as described in A above with slight modification with vehicle (E Control), everolimus (E) 0.5mg/kg and 1mg/kg o.g. daily continuously, pasireotide LAR vehicle subcut once (P LAR control) and pasireotide LAR 20mg/kg once subcut (P LAR) or the combination of pasireotide and everolimus.

Pasireotide and everolimus, as single agents, achieved modest activity in the preceding i*n vitro* and *in vivo* experiments. To assess whether the combination of both agents will be more effective than each agent alone, we tested the combination of pasireotide and everolimus and compared to the single agent using both BCPAP and TPC-1 thyroid cancer xenograft models. Consistent with the *in vitro* results, there was additive effect with the combination of everolimus and pasireotide in inhibiting tumor growth in the TPC-1 xenograft (Fig 5A).

There was only modest growth inhibition observed with single agent everolimus (p = 0.28) and single agent pasireotide (p = 0.25) compared to their respective controls. The combination of both agents achieved a statistically significant reduction in tumor volume compared to control (p = 0.04). There was no significant additive toxicity of the combination as indicated by the comparable weights of tumor-bearing mice treated with vehicle, single agent or the combination regimens. Overall, the efficacy was more modest in the BCPAP xenograft and the trend of greater antitumor efficacy with the combination in comparison to the single agent did not reach statistical significance (p = 0.14), (Fig 5B).

## Discussion

Downstream signaling through the mTOR and somatostatin signaling pathways is important for physiologic proliferation and function of the normal thyroid gland.[13, 25, 26] These pathways remain essential following malignant transformation of the thyroid epithelium.[8] There is therefore a valid biological rationale to anticipate that therapeutic targeting of these pathways will result in preclinical and ultimately clinical efficacy. Indeed, prior studies showed antiproliferative activity of everolimus in preclinical models of thyroid cancer[27, 28]. Moreover, the negative modulation of the PI3K/AKT pathway induced by somatostatin binding to its receptors can counteract the reactive activation of AKT following everolimus therapy, which is one of the mechanisms postulated to limit the clinical efficacy of mTOR inhibitors [29–31]. It is thus plausible that the increased vertical blockade and lack of AKT activation with the combination of a somatostatin analogue and an mTOR inhibitor will result in enhanced effect over each alone. For this reason, prior works evaluated the potential improved efficacy of octreotide and other somatostatin analogues, in combination with rapamycin in various types of endocrine tumors such as pancreatic neuroendocrine tumor, pheochromocytoma and pituitary adenoma. The results of these studies have been discordant in part because of the variability and diversity of SSTR receptor subtypes in different tumor types.[17, 32] Pasireotide is a multi-receptor ligand somatostatin analogue that binds with high affinity to four of the five SSTR subtypes (subtypes 1, 2, 3 and 5) and was designed to have greater antiproliferative potency over octreotide, which only binds to two SSTR subtypes, 2 and 5.[19] Pasireotide is therefore expected to achieve improved and consistent efficacy in endocrine cancers. Whether and how pasireotide can potentiate everolimus, a clinically relevant mTOR inhibitor, in thyroid cancer of follicular epithelium origin has not been studied previously. In this study, we evaluated the preclinical activity of everolimus and pasireotide in thyroid cancer cell lines of follicular epithelium origin and corresponding representative tumor xenografts.

As anticipated, we observed a modest degree of *in vitro* activity of both everolimus and pasireotide with variability in the sensitivity of the cell lines to each of the two agents (Figs 1 and 2). Comparatively, everolimus appeared more effective than pasireotide in short term and long term *in vitro* cytotoxicity assays. Consistent with prior reports, everolimus showed antiproliferative activity[27, 28]. However, the effect was mainly cytostatic against thyroid cancer cells with no evidence of inducing apoptotic cell death as assessed by caspase 3 cleavage (Fig 3 **and Figures J and K in** S1 File**)**. As expected, the addition of pasireotide to everolimus lead to enhanced vertical blockade of the PI3K/AKT/mTOR pathway as demonstrated by the greater reduction in pS6 expression with combined treatment over everolimus alone (Fig 3 **and Figures R and S in** S1 File). This enhanced pathway blockade did not, however, result in significant increase in cell growth inhibition in *in vitro* cytotoxicity assays (Figs 1 and 2). In *in vivo* experiments using TPC-1 xenograft models, the combination of pasireotide with everolimus led to a greater degree of tumor growth inhibition over everolimus alone (Fig 5A). This is consistent with earlier works showing a positive interaction between rapamycin and a first generation somatostatin analogue, octreotide in pituitary tumors. Several complementary mechanisms probably explain the cooperative interaction between everolimus and pasireotide. For instance, rapalogs inhibit cellular proliferation by inactivating mTOR, which results in dephosphorylation of its effectors, 4E-BP1 and S6K1 with consequent inhibition of cap-dependent translation and cell growth [33]. In addition to this direct effect on cellular growth, rapalogs can also inhibit tumor angiogenesis. Similarly, the binding of somatostatin and its pharmacological analogues to SSTR directly inhibits cell proliferation through a negative modulation of the PI3K/AKT/mTOR pathway and the induction of p27Kip1, a cyclin-dependent kinase inhibitor, by G protein-coupled SSTR2 through the activation of tyrosine phosphatase SHP-1 [34]. Additionally, pasireotide and other somatostatin analogues have indirect antitumor effect by suppressing the secretion of growth factors and angiogenesis [35].

SSTR is frequently expressed in thyroid cancer, with SSTR1 being the most commonly expressed in up to 88.8% of cases while SSTR2 is expressed in 44% of cases.[8, 12] We confirmed that all SSTR subtypes including SSTR4 are expressed in our panel of thyroid cancer cell lines both by Western blot and by immunofluorescence that is able to detect native receptor expression on the intact cell membranes (Fig 3). SSTR2 showed the highest intensity of staining of all the 5 subtypes by immunofluorescence in 5 of the 6 cell lines. However, despite the demonstration of high SSTR expression in our cell line panel, pasireotide had negligible *in vitro* activity and only modest activity against thyroid cancer xenografts *in vivo*. The limited efficacy of pasireotide may reflect the unique biology of pasireotide binding to SSTR2 subtype, which has been shown to be critical for the growth inhibitory effects of somatostatin signaling pathway in various tumor types [36, 37]. Following somatostatin agonist stimulation, full dephosphorylation and recovery of active SSTR2 depends on receptor internalization, which occurs with first generation analogues, octreotide and lanreotide, but not with pasireotide [6, 38]. This lack of receptor internalization could in part be responsible for the negligible *in vitro* anti proliferative activity of pasireotide in these cell lines where SSTR2 is the predominant receptor subtype. Consistent with this observation, there was no significant correlation between SSTR2 expression and pasireotide (CC: 0.36) or everolimus (CC: 0.11) in vitro efficacy whereas SSTR3 showed strong correlation with pasireotide activity (CC: 0.81) while SSTR1 expression was strongly correlated with everolimus activity (CC: 0.85).

In conclusion, our preclinical findings provide a rationale for combined targeting of mTOR and somatostatin signaling pathways as a promising approach for thyroid cancer therapy and provide further support for the ongoing exploration of this combination strategy in the clinic for the treatment of thyroid cancer and other neuroendocrine tumors.

## Supporting information

S1 Fig Representative pictures of colony formation assay in TPC-1, Cal-62 and BCPAP. (TIF)

S1 File Original images of Western blots for Figure A—SSTR1, Figure B—SSTR2, Figure C- SSTR3, Figure D—SSTR4, Figure E—SSTR5, Figure F - 4eBP1, Figure G—Actin, Figure H–Actin2, Figure I—AKT, Figure J—Caspase3, Figure K—Cleaved Caspase3, Figure L—eIF4e, Figure M—mTOR, Figure N- p-4eBP1, Figure O—p-AKT, Figure P—p-eIF4e, Figure Q—p-mTOR, Figure R—p-S6 and Figure S—S6and.
(PPTX)
