# Supplementary figures and images for: Evaluation of preclinical efficacy of everolimus and pasireotide in thyroid cancer cell lines and xenograft models
Source: PLoS One. 2019 Feb 26;14(2):e0206309. doi: 10.1371/journal.pone.0206309 (PMC6390992; doi:10.1371/journal.pone.0206309)

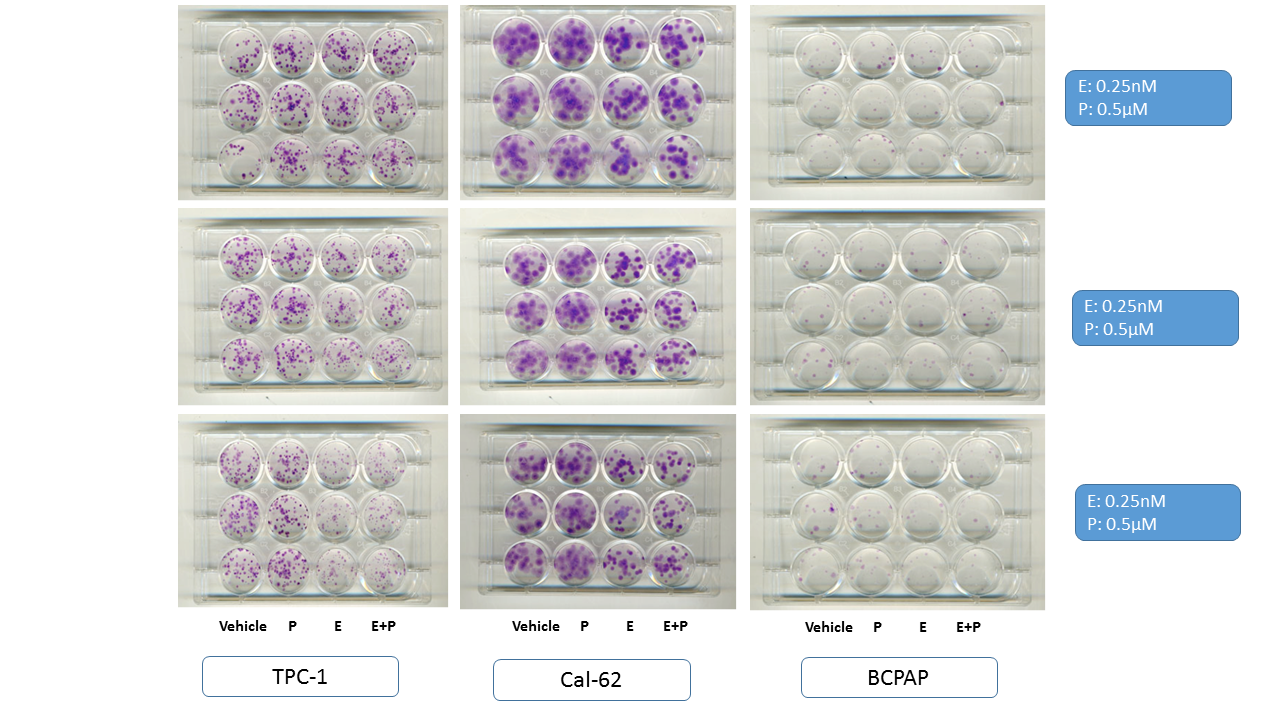

Supplement: S1 Fig — (TIF) [file pone.0206309.s001.tif]
