# Supplementary material for: Evaluation of preclinical efficacy of everolimus and pasireotide in thyroid cancer cell lines and xenograft models
Source: PLoS One. 2019 Feb 26;14(2):e0206309. doi: 10.1371/journal.pone.0206309 (PMC6390992; doi:10.1371/journal.pone.0206309)
Supplement: S1 File — Original images of Western blots for Figure A—SSTR1, Figure B—SSTR2, Figure C- SSTR3, Figure D—SSTR4, Figure E—SSTR5, Figure F - 4eBP1, Figure G—Actin, Figure H–Actin2, Figure I—AKT, Figure J—Caspase3, Figure K—Cleaved Caspase3, Figure L—eIF4e, Figure M—mTOR, Figure N- p-4eBP1, Figure O—p-AKT, Figure P—p-eIF4e, Figure Q—p-mTOR, Figure R—p-S6 and Figure S—S6and. (PPTX) [file pone.0206309.s002.pptx]

## Slide 1
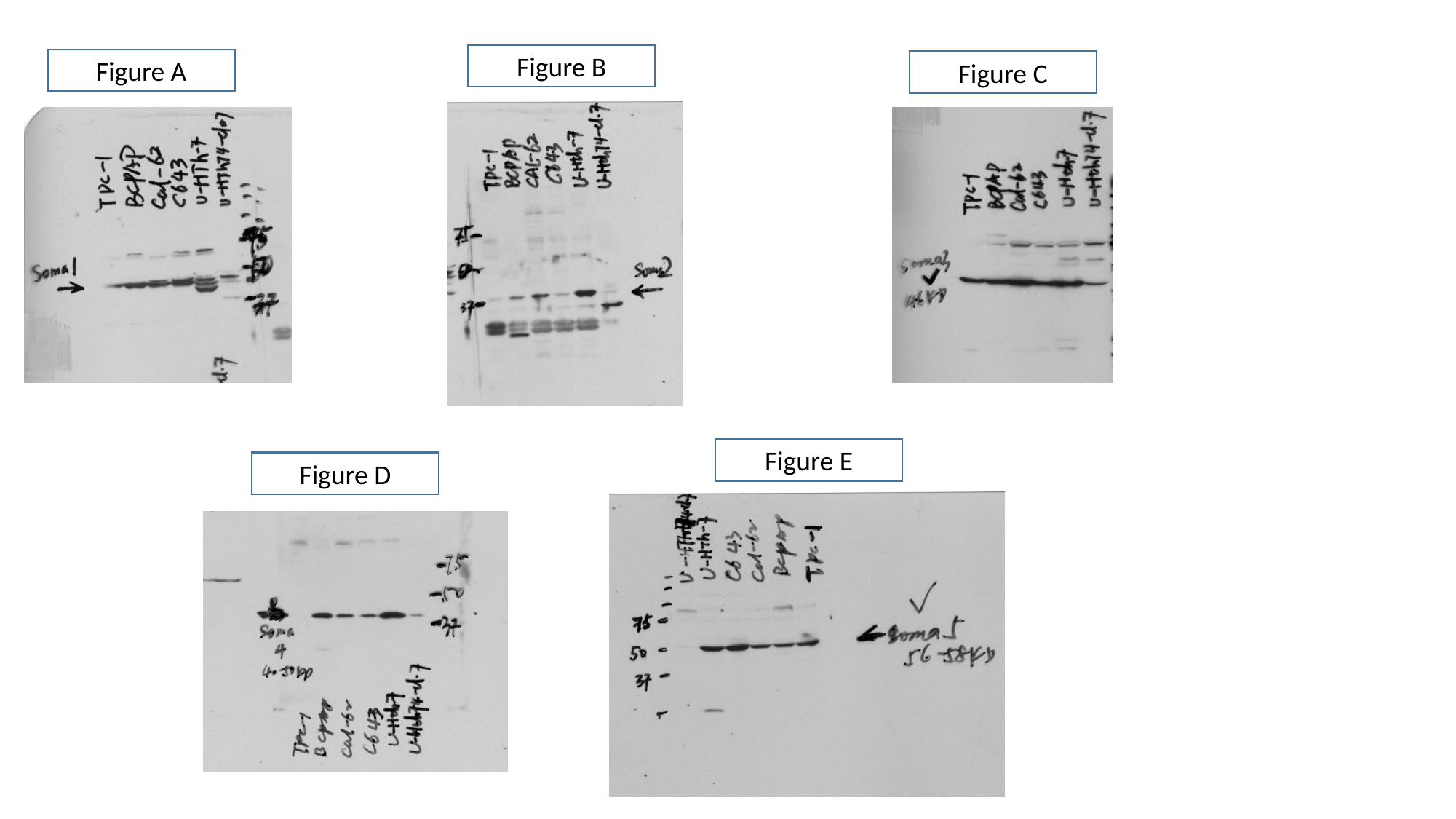

Figure B
Figure A
Figure C
Figure E
Figure D

## Slide 2
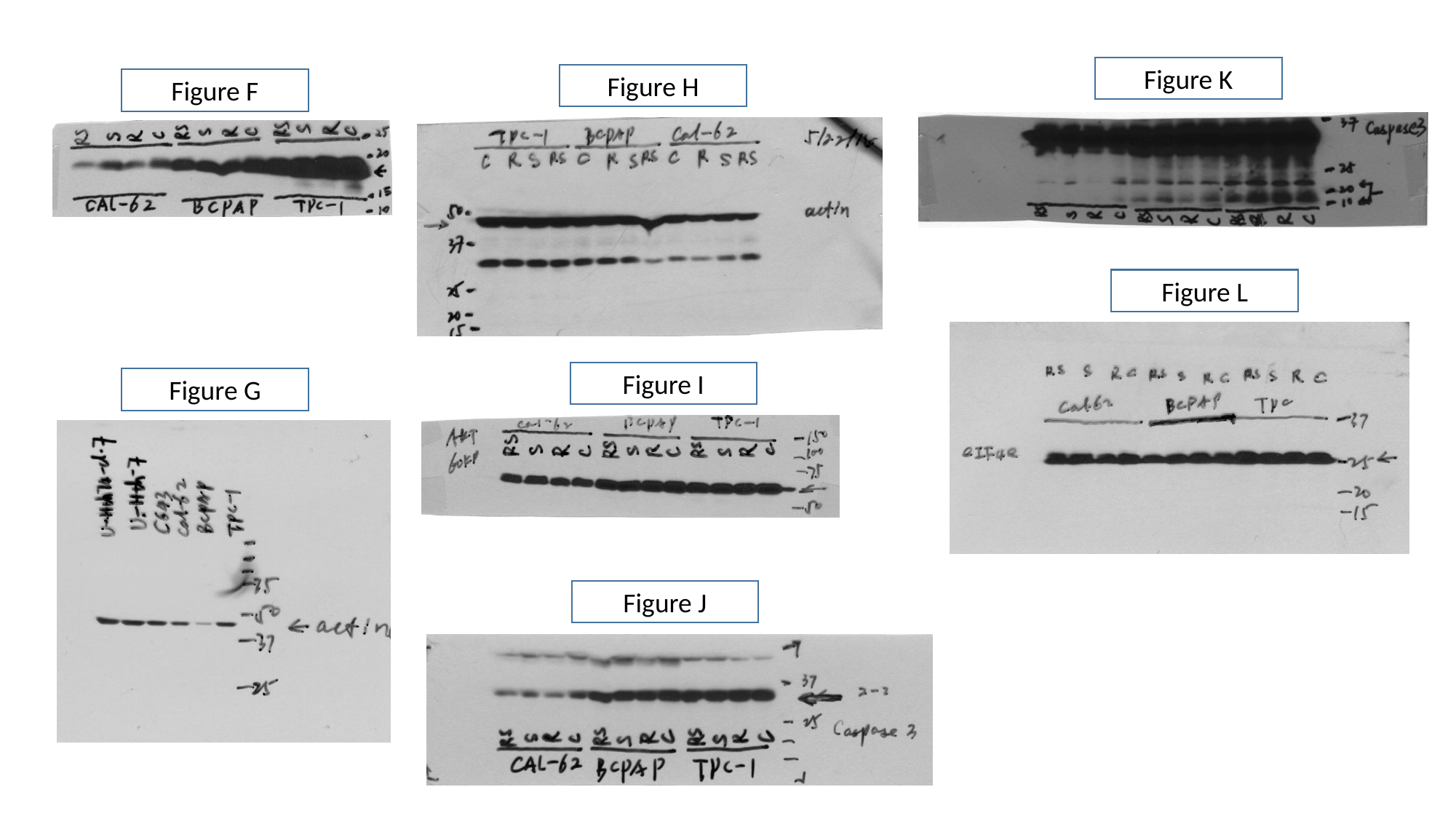

Figure K
Figure H
Figure F
Figure L
Figure I
Figure G
Figure J

## Slide 3
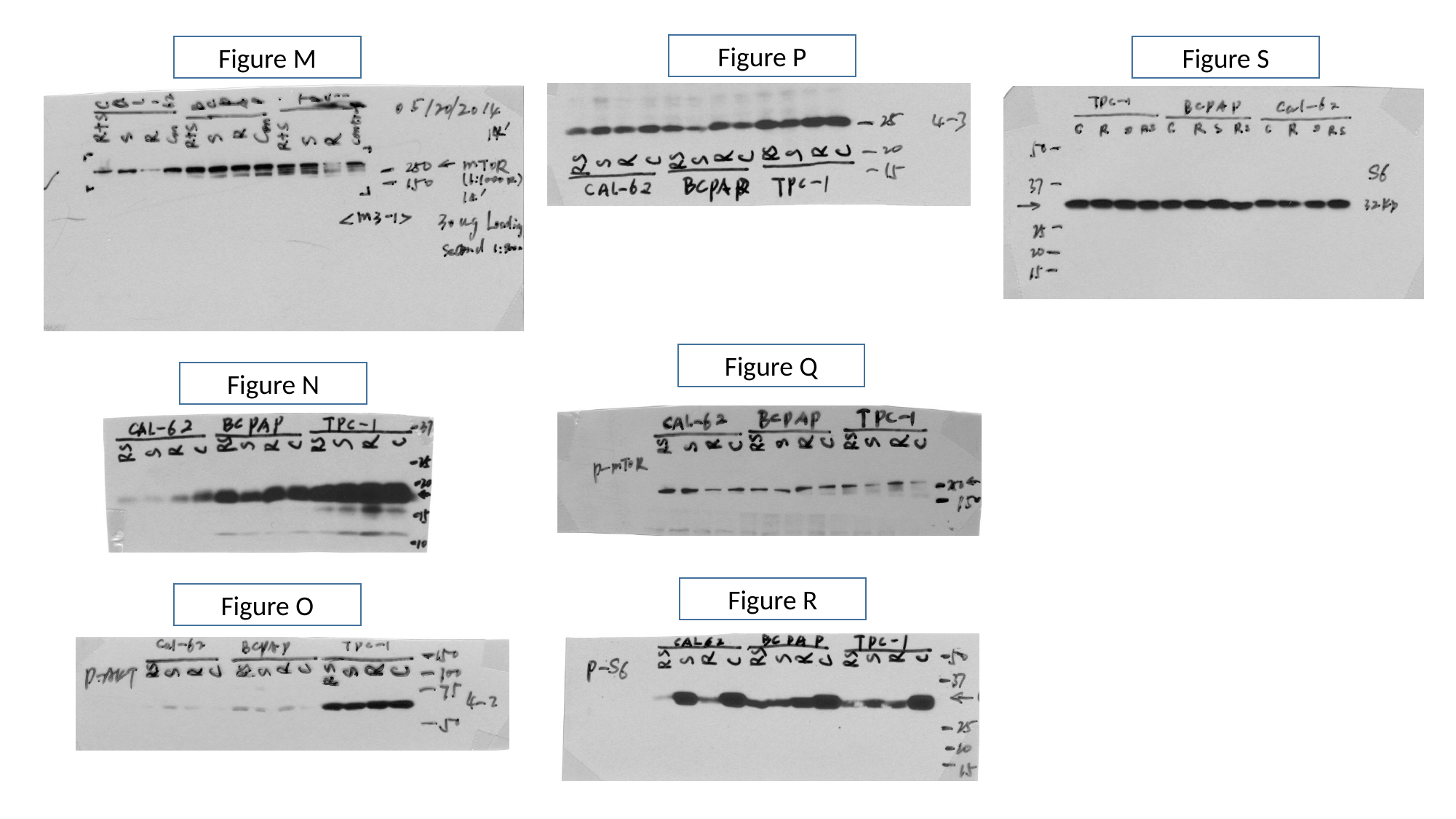

Figure P
Figure M
Figure S
Figure Q
Figure N
Figure R
Figure O
